# Knowledge, attitude, and practice of artificial intelligence among doctors and medical students in Syria: A cross-sectional online survey

**DOI:** 10.3389/frai.2022.1011524

**Published:** 2022-09-29

**Authors:** Sarya Swed, Hidar Alibrahim, Nashaat Kamal Hamdy Elkalagi, Mohamad Nour Nasif, Mohammed Amir Rais, Abdulqadir J. Nashwan, Ahmed Aljabali, Mohamed Elsayed, Bisher Sawaf, Mhd Kutaiba Albuni, Elias Battikh, Leena Abdelwahab Mohamed Elsharif, Safaa Mohamed Alsharief Ahmed, Eman Mohammed Sharif Ahmed, Zain Alabdeen Othman, Ahmad Alsaleh, Sheikh Shoib

**Affiliations:** ^1^Faculty of Medicine, Aleppo University, Aleppo, Syria; ^2^Internal and Tropical Medicine, Faculty of Medicine, Arish University, Al Arish, Egypt; ^3^Faculty of Medicine of Algiers, University of Algiers, Algiers, Algeria; ^4^Nursing Department, Hamad Medical Corporation, Doha, Qatar; ^5^Faculty of Medicine, Jordan University of Science and Technology, Irbid, Jordan; ^6^Department of Psychiatry and Psychotherapy III, University of Ulm, Ulm, Germany; ^7^Department of Psychiatry, School of Medicine and Health Sciences, Carl von Ossietzky University Oldenburg, Oldenburg, Germany; ^8^Department of Internal Medicine, Hamad Medical Corporation, Doha, Qatar; ^9^Faculty of Medicine, Red Sea University, Port Sudan, Sudan; ^10^Faculty of Medicine, Shendi University, Shendi, Sudan; ^11^Department of Obstetrics and Gynecology, Nile Valley University, Khartoum, Sudan; ^12^Faculty of Dentistry, Albaath University, Homs, Syria; ^13^Faculty of Medicine, Damascus University, Damascus, Syria; ^14^JLNM Hospital, Rainawari, Srinagar, J&K, India; ^15^Directorate of Health Services, Kashmir, J&K, India

**Keywords:** knowledge, attitude, practice, artificial intelligence, medical student, doctor, Syria

## Abstract

Artificial intelligence has been prevalent recently as its use in the medical field is noticed to be increased. However, middle east countries like Syria are deficient in multiple AI implementation methods in the field of medicine. So, holding these AI implementation methods in the medical field is necessary, which may be incredibly beneficial for making diagnosis more accessible and help in the treatment. This paper intends to determine AI's knowledge, attitude, and practice among doctors and medical students in Syria. A questionnaire conducted an online cross-sectional study on the google form website consisting of demographic data, knowledge, and perception of AI. There were 1,494 responses from both doctors and medical students. We included Syrian medical students and doctors who are currently residing in Syria. Of the 1,494 participants, 255 (16.9%) are doctors, while the other 1,252 (83.1%) are undergraduate medical students. About 1,055 (70%) participants have previous knowledge about AI. However, only 357 (23.7%) participants know about its application in the medical field. Most have shown positive attitudes toward its necessity in the medical field; 689 (45.7%) individuals strongly agree, and 628 (41.7%) agree. The undergraduate students had 3.327 times more adequate knowledge of AI than students in the first year. In contrast, the undergraduate 6th-year students had 2.868 times the attitude toward AI higher than students in the first year. The residents and assistant professors had 2.371 and 4.422 times the practice of AI higher than students, respectively. Although most physicians and medical students do not sufficiently understand AI and its significance in the medical field, they have favorable views regarding using AI in the medical field. Syrian medical authorities and international organizations should suggest including artificial intelligence in the medical field, particularly when training residents and fellowship physicians.

## Background

Artificial intelligence is a software system that attempts to simulate human intellect by using data sources to make independent decisions or assist humans in making decisions (He et al., 2019; Hashimoto et al., 2020). It is a general term that includes machine learning, representation learning, deep learning, and natural language processing. Artificial intelligence is a field of computer science that can analyze large amounts of data. However, it is not only related to computer science but extends into many areas such as medicine, philosophy, psychology, linguistics, and statistics (He et al., 2019). In medicine, it has contributed to treating several illnesses and decreased many mistakes in diagnosis and follow-up (Patel et al., 2009; Wang et al., 2019). It has increased recently, expanding from research to application in various sectors (Alami et al., 2020). High-income countries have provided substantial financial support for AI research, especially in the medical field. In low-income countries like Syria, there are no serious plans to implement AI, furthermore a lack of research about it (WHO, 2017). According to WHO, by 2035, there will be a shortage of nearly 12.9 million health care workers worldwide (WHO, 2017). In addition, in the last 60 years, artificial intelligence (AI) has seen significant development. However, machine learning use in low-resource countries has remained comparatively slight (Wahl et al., 2018; Triantafyllidis and Tsanas, 2019). Machine learning has been used in many medical sectors, including Diabetes, cancer, cardiology, mental health, radiology, and others (Thrall et al., 2018). Radiology provides a more direct pathway for AI because it offers digitally coded images that can be easily converted into computer language (Niazi et al., 2019). Several studies have suggested the critical role of AI in pathology (Souza Filho et al., 2020). It can provide image-based diagnosis options and increase the pathologist's understanding of microscopic slides by inserting electronic slides and computer-aided diagnostic procedures. Machine learning and deep learning have been used in cardiology to identify patients at risk of rapid coronary plaque progression, anticipate the chance of a heart attack, and determine prognosis in pulmonary hypertension patients (Bonderman, 2017; Wozniacka et al., 2021). Artificial intelligence is becoming more relevant than dermatologists, especially in diagnosing skin lesions from clinical and dermoscopic images (Niel and Bastard, 2019). AI techniques are developing in ophthalmology, particularly in diabetic retinopathy, age-related macular degeneration, and retinopathy of prematurity (Hashimoto et al., 2020). It was applied in nephrology to raise medical management, hemodialysis treatments, and transplant patient follow-up (Hessler and Baringhaus, 2018). It has a wide application in drug design, including *de novo* chemical compound and peptide design, as well as synthesis planning (Chan and Zary, 2019). The healthcare sector seems to be well-suited for transformation by AI. By transcribing notes, inputting and arranging patient data onto portals (such as EPIC), and diagnosing patients, AI systems might possibly spare up time for busy doctors and serve as a second opinion for them. In addition, patients may benefit from the availability of alternatives to prescribed medications and follow-up treatment from artificially intelligent systems. Aside from the world's main metropolitan centers, AI has the potential to diagnose patients remotely, enabling the expansion of medical services into rural regions. There is still much work to be done, but the future of AI in healthcare is bright and hopeful. AI may help lessen some of the drawbacks of traditional methods of diagnosis and treatment, including the risk of errors because of burnout in the medical field and other psychological impacts, the need to examine many patients quickly, the occasional inaccuracy of the diagnosis, and patients' anxiety when confronted by a clinical doctor. However, there are also unbelievable preconceptions of what AI is capable of and how the future of the healthcare sector will pan out, such as the assumption that AI will eventually replace doctors and that programming skills are required to use AI effectively. It was necessary to introduce AI in medical education because it can provide specific feedback to support learning and better understand AI algorithms (Rabaan et al., 2022). There were significant obstacles to integrating AI in the healthcare sector after the Syrian conflict, including the disintegration of healthcare services, the devastation of hospitals, the migration of healthcare staff, and the fall in medical education and training. This study aims to identify the level of doctors' and medical students' know carried perceptions of AI and its implications in Syria and evaluate their understanding of various AI practices.

## Method

### Study design and sample size

During the period 7 April to 21 May 2022, we distributed a web-based survey to medical students and physicians in Syria using social media apps (WhatsApp, Facebook, and Messenger) and email. This research was a cross-sectional study. Based on prior research conducted in Pakistan, a questionnaire was translated from the Pakistani language to Arabic, developed, investigated, and confirmed to be accurate for Syrian students and doctors (Ahmed et al., 2022). Responses were anonymous without any identifying data, and only the principal investigator had access to the data. A convenience sampling technique was used to pick the sample population. We conducted an experimental survey on 30 participants before distributing the questionnaire to test the usability and technical performance of the online survey. After that, the questionnaire was tested on 50 people as part of a pilot study to confirm its validity and reliability. The tool's internal consistency of the used sub-scales was shown by Cronbach's alpha values, ranging from 0.7 to 0.8 (Knowledge = 0.795, practice = 0.702, and Attitude = 0.663). Participants had the option to go back and modify their replies. The duplicate entries were cleaned up and only completed responses were considered. Inclusion criteria were responders being medical students and doctors conducting the study. Exclusion criteria were non-medical responders and an incomplete survey. Calculator.net's sample size estimator, accessible at “https://www.calculator.net/sample-size-calculator.html”, was used to determine the sample size. The population of Syria is expected to reach over 18 million people in 2019, according to statistics from the UN. Based on that, we performed a statistical power analysis to calculate the sample size. The suggested sample size was 385 with a design effect of 50%, a margin of error of 0.05, and a confidence level of 95%. Participants were encouraged to complete the online survey on the Google form, which had 1,538 total answers. The total sample size was 1,484 after 24 people declined to complete the questionnaire.

### Measurements

#### Demographic information

The questionnaire consists of age, gender, qualification level, rank, and university year for the undergraduate participants.

#### Knowledge toward artificial intelligence

This sub-scale has seven questions about the general knowledge of AI, including knowledge of artificial intelligence machine learning, AI in the medical field, AI in radiology, AI in pathology, and AI during the training for post-graduate doctors (for the statistical analysis, yes = 1, no = 0 & Good knowledge is upper than 3 points).

#### Attitude toward artificial intelligence

This sub-scale has ten questions about the attitude toward AI, including the necessity of AT in the medical field, training, assessment, diagnosis, radiology, pathology, and its importance during the COVID19 (for the statistical analysis, Don't know, disagree or strongly disagree = 0, agree or strongly agree = 0 & Good attitude is upper than 5 points).

#### Practice toward artificial intelligence

This sub-scale has seven questions about the practice of AI, including if the doctor has inserted the AI in the medical field and the intention of conducting this technique during the training (for the statistical analysis, yes = 1, No, never applied = 0 & Good practice is upper than 2 points).

### Ethical approval

All participants could withdraw from the cross-sectional research at any time, and participation was completely voluntary. The participant could not be identified since the study did not provide names or emails. Each participant's identity was therefore wholly protected during the investigation. The University of Aleppo's ethics committee granted its permission and gave the research the go-ahead, and the Helsinki Declaration carried out the study.

### Statistical analysis

The data was analyzed using SPSS version 25.0. The frequencies of different variables were shown using frequency tables. The internal consistency of the scale was determined using Cronbach's α coefficient. The Chi-square test was used to examine the statistical correlation between the categorical variables. A *p*-value of less than 0.05 indicated that the association was significant. Mann-Whitney and Kruskal-Wallis tests were used depending on the data normality. In addition, univariate logistic regression was carried out to predict the outcome measurements of artificial intelligence, such as the knowledge, attitude, and practice from the baseline characteristics of the study population. The unadjusted odds ratios and their respective 95% confidence intervals were used in the regression.

## Results

As shown in the baseline characteristics of the study population in Table 1, there are 255 (16.9%) who are a graduate or postgraduate master's or Ph.D. doctors, while medical students resemble about 1,252 (83.1%) of the total participants, most of them aged between 21 and 30 years, 1,452 (97.2%). Most participants were male, Gender 779 (52%), and most undergraduate participants were from the sixth year of medical school 476 (31.6).

**Table 1:** Baseline characteristics of the study population.

		**Frequency**	**Percentage %**
Age	21–30	1,452	97.2%
	31–40	25	1.7%
	41–50	4	0.3%
	51–60	8	0.5%
	60<	5	0.3%
Gender	Males	779	52.0%
	Females	718	48.0%
Qualification level	Undergraduate	1,252	83.1%
	Graduate	255	16.9%
If undergraduate, then which university year?	First-year	126	8.4%
	Second year	114	7.6%
	third year	145	9.6%
	fourth year	82	5.4%
	fifth year	286	19.0%
	sixth year	476	31.6%
	Graduate	278	18.4%
If graduate, then current status	Student	1,277	84.7%
	Resident	182	12.1%
	Medical practitioner	24	1.6%
	Senior house officer	7	0.5%
	House officer	17	1.1%
If postgraduate, specify the rank:	Student	1,284	85.2%
	Resident	193	12.8%
	Senior registrar	12	9.8%
	Assistant professor	12	9.8%
	Associate professor	1	9.1%
	Professor	5	9.3%

### Knowledge of AI

As shown in Table 2, the mean score of Knowledge of AI was 1.82 ± 1.83. Regarding Knowledge of AI, individuals were questioned about the basic concept of AI, its subtypes, i.e., machine learning (ML) and deep learning (DL), and its applications. It was observed that 1,055 (70%) had a basic concept of AI, but only 523 (34.7%) had Knowledge about ML and DL, and only 357 (23.7%) had Knowledge about its applications. In contrast, 452 (30%) individuals had no knowledge about the basic concept of AI, 984 (65.3%) had no knowledge about ML and DL, and 1,150 (65.3%) were unaware of any application of AI in the medical field. Only 320 (21.2%) individuals were aware of the application of AI in radiology, and only 261 (17.3%) knew about AI application in pathology.

**Table 2:** Descriptive statistics for knowledge of artificial intelligence.

		**Frequency**	**Percentage %**
Do you know what artificial intelligence is?	No	452	30.0%
	Yes	1,055	70.0%
Do you know about machine learning and deep learning (subtypes of AI)?	No	984	65.3%
	Yes	523	34.7%
Do you know about any application of AI in the medical field?*	No	1,150	76.3%
	Yes	357	23.7%
Have you ever been taught about Artificial intelligence in medical school?*	No	1,345	89.3%
	Yes	162	10.7%
Do you know about the application of AI in radiology?	No	1,187	78.8%
	Yes	320	21.2%
Do you know about the application of AI in the pathology field?*	No	1,246	82.7%
	Yes	261	17.3%
If you are a PGR, does your training include a curriculum regarding AI?	No	1,441	95.6%
	Yes	66	4.4%

* *P*-value < 0.05.

There were significant differences in the knowledge score as a continuous dependent variable about Gender (*P* < 0.001) and qualification level (*P* < 0.001), in which the females and the graduate doctors have higher knowledge than others, but not for the age groups (*P* = 0.297), as shown in Table 3.

**Table 3:** Knowledge, attitude and practice score of AI in medical students and doctors.

	**Gender**	**Mean**	**Std. Deviation**	***p*-value**
Knowledge of artificial intelligence	Male	1.6508	1.77494	0.000
	Female	2.0195	1.87166	
	Total	1.8277	1.83065	
Practice of AI	Male	1.9114	1.00697	0.968
	Female	1.9136	1.11828	
	Total	1.9125	1.06146	
Attitude toward AI	Male	6.2169	2.08259	0.001
	Female	5.8607	2.22640	
	Total	6.0461	2.15939	
	Qualification level			
Knowledge of artificial intelligence	Undergraduate	1.7093	1.72909	0.000
	Graduate	2.3686	2.18743	
	Total	1.8208	1.83076	
Practice of AI	Undergraduate	1.8147	0.91833	0.000
	Graduate	2.3725	1.50014	
	Total	1.9091	1.06013	
Attitude toward AI	Undergraduate	6.1134	2.12929	0.003
	Graduate	5.6784	2.28452	
	Total	6.0398	2.16172	
	Age			
Knowledge of artificial intelligence	21–30	1.8079	1.81528	0.297
	31–40	2.3600	2.32522	
	41–50	3.0000	2.70801	
	51–60	2.3750	2.26385	
	60<	1.4000	1.51658	
	Total	1.8220	1.82904	
Practice of AI	21–30	1.9001	1.02838	0.004
	31–40	2.4800	1.78232	
	41–50	2.5000	2.08167	
	51–60	2.3750	2.26385	
	60<	0.8000	0.83666	
	Total	1.9103	1.06077	
Attitude toward AI	21–30	6.0861	2.13407	0.000
	31–40	5.0800	2.39653	
	41–50	4.2500	3.40343	
	51–60	4.3750	2.44584	
	60<	3.4000	3.20936	
	Total	6.0462	2.15908	

Table 4 demonstrates how Knowledge of AI can differ depending on different baseline variables. Qualification level, undergraduate year, graduate current status, and postgraduate rank significantly differs in the proportion of good Knowledge.

**Table 4:** Knowledge of AI based on gender, age, qualification level, professional, current status and rank of the doctors.

		**Knowledge of Artificial intelligence**				
		**Poor**		**Good**		***P*-value**
		**Frequency**	**Percentage**	**Frequency**	**Percentage**	
Age	21–30	1,218	80.8%	234	15.5%	0.519
	31–40	18	1.2%	7	0.5%	
	41–50	3	0.2%	1	0.1%	
	51–60	6	0.4%	2	0.1%	
	60<	4	0.3%	1	0.1%	
Gender	Male	661	43.9%	118	7.8%	0.142
	Female	589	39.1%	129	8.6%	
Qualification level	Undergraduate	1,069	70.5%	183	12.1%	0.00002*
	Graduate	190	12.9%	65	4.4%	
If undergraduate, then which professional?	1st professional	112	7.4%	14	0.9%	0.000*
	2nd professional	96	6.4%	18	1.2%	
	3rd professional	130	8.6%	15	1%	
	4th professional	59	3.9%	23	1.5%	
	5th professional	230	15.3%	56	3.7%	
	6th professional	422	28.0%	54	3.6%	
	Graduate	210	13.9%	68	4.5%	
If graduate, then current status	Student	1,089	72.3%	188	12.5%	0.000279
	Resident	138	9.2%	44	2.9%	
	Medical practitioner	16	1.1%	8	0.5%	
	Senior house officer	4	0.8%	5	0.3%	
	House officer	12	0.3%	3	0.2%	
If postgraduate, specify the rank:	Student	1,096	72.7%	188	12.5%	0.000002
	Resident	146	9.7%	47	3.1%	
	Senior registrar	9	0.6%	3	0.2%	
	Assistant professor	7	0.5%	5	0.3%	
	Associate professor	0	0.0%	1	0.1%	
	Professor	1	0.1%	4	0.3%	

* *P*-value < 0.05.

The proportion of good Knowledge was 183 (12.1%) compared to 65 (4.4%) among the undergraduate and graduate participants. Undergraduate fifth-year medical students were the most who had an excellent knowledge 56 (3.7%). Compared to the residents and other categories, graduate student participants had a higher proportion of good knowledge 188 (12.5%), and the same for postgraduate students compared to professors and different types 188 (12.5%).

The prediction of adequate Knowledge of AI among the study sample depending on the demographic variables is given in Table 5. It was observed that the level of undergraduate medical students' 4th and 5th years was the only significant factor affecting their Knowledge of AI. The undergraduate students had 3.327 times more adequate knowledge of AI than students in the first year.

**Table 5:** Binary logistic regression between baseline characteristics of the study population and the knowledge of artificial intelligence^*^.

	**Categories**	***P*-value**	**Odds ratio**	**Lower**	**Upper**
Age	21–30	0.997	Reference
	31–40	0.830	1.125	0.830	1.125
	41–50	0.999	0.000	0.999	0.000
	51–60	0.722	1.373	0.722	1.373
	60<	0.891	1.174	0.891	1.174
Level of education	undergraduate	Reference		
	graduate	0.276	2.366	0.503	11.129
Gender	Male	Reference			
	Female	0.256	1.182	0.886	1.576
If undergraduate, then which professional?	1st professional	0.001	Reference		
	2nd professional	0.299	1.508	0.299	1.508
	3rd professional	0.978	0.989	0.978	0.989
	4th professional	0.002	3.327	0.002	3.327
	5th professional	0.025	2.092	0.025	2.092
	6th professional	0.673	1.150	0.673	1.150
	Graduate	0.692	0.724	0.692	0.724
If postgraduate, specify the rank	Student	0.569	Reference		
	Resident	0.231	1.639	0.231	1.639
	Senior registrar	0.528	1.652	0.528	1.652
	Assistant professor	0.053	3.784	0.053	3.784
	Associate Professor	1.000	-	-	-
	Professor	0.221	5.251	0.370	74.602
Constant	0.000	0.107			

* The logistic regression model was statistically significant, X2 (7) = 58.33, p-value = 0.000, Hosmer and lemeshow test: 15.73(P-value = 0.028), The model explained 0.065 Nagelkerke R Square of the variance in knowledge of artificial intelligence among doctors and medical students in Syria.

### Attitude toward AI

The mean score of attitudes toward AI was 6.03 ± 2.16. Regarding the attitude toward AI in the health sector, individuals were questioned about the necessity of AI in the medical field, 689 (45.7%) individuals strongly agreed, and 628 (41.7%) agreed. Regarding the opinion that AI aids practitioners in early diagnosis and assessment of disease severity, 558 (37%) strongly agree, and 690 (45.8%) agree. The idea that AI can replace the physician in the future, 127 (8.4%) strongly agree, and 97 (13.1%) agree. Individuals believe that AI is essential in radiology and pathology, as 445 (29.5) and 396 (26.3%) strongly agree. Out of all, 661 (43.9%) agree that introducing AI is essential in the current COVID 19 pandemic. While 684 (45.4%) disagree that AI would be a burden for practitioners regardless of the pandemic. Regarding the budget allocated for AI to be used in the current COVID 19 pandemics, most individuals agree 648 (43%). Most individuals disagree that AI would increase the percentage of errors in diagnosis 496 (32.9%), as shown in Table 6.

**Table 6:** Descriptive statistics for attitude toward artificial intelligence.

		**Frequency**	**Percentage %**
Do you believe AI is essential in the medical field?	agree	628	41.7%
	strongly agree	689	45.7%
	Don't Know	151	10.0%
	disagree	33	2.2%
	strongly disagree	6	0.4%
Do you think AI should be included in the curriculum in medical school as well as specialist training?	agree	680	45.1%
	strongly agree	606	40.2%
	Don't Know	128	8.5%
	disagree	76	5.0%
	strongly disagree	17	1.1%
Do you think that AI aids practitioners in early diagnosis and assessment of the severity of disease?	agree	690	45.8%
	strongly agree	558	37.0%
	Don't Know	186	12.3%
	disagree	60	4.0%
	strongly disagree	13	0.9%
Do you believe that AI will replace physicians in the future?	agree	197	13.1%
	strongly agree	127	8.4%
	Don't Know	327	21.7%
	disagree	551	36.6%
	strongly disagree	305	20.2%
Do you believe AI is very essential in the field of radiology?	agree	660	43.8%
	strongly agree	445	29.5%
	Don't Know	342	22.7%
	disagree	51	3.4%
	strongly disagree	9	0.6%
Do You believe AI is essential in the field of Pathology?	agree	711	47.2%
	strongly agree	396	26.3%
	Don't Know	343	22.8%
	disagree	52	3.5%
	strongly disagree	5	0.3%
Do you think the introduction of AI is essential in the current COVID 19 pandemic?	agree	661	43.9%
	strongly agree	365	24.2%
	Don't Know	376	25.0%
	disagree	87	5.8%
	strongly disagree	18	1.2%
Do you believe AI would be a burden for practitioners?	agree	154	10.2%
	strongly agree	114	7.6%
	Don't Know	387	25.7%
	disagree	684	45.4%
	strongly disagree	168	11.1%
Do you believe the budget should be allocated for AI to be used in the current COVID 19 pandemics?	agree	648	43.0%
	strongly agree	366	24.3%
	Don't Know	328	21.8%
	disagree	141	9.4%
	strongly disagree	24	1.6%
Do you believe AI would increase the percentage of errors in diagnosis?	agree	273	18.1%
	strongly agree	134	8.9%
	Don't Know	488	32.4%
	disagree	496	32.9%
	strongly disagree	116	7.7%

There were significant differences in the attitude score as a continuous dependent variable about Gender (*P* < 0.001), qualification level (*P* = 0.003), and age groups (*P* < 0.001), which the males, the undergraduate, and 21–30 years old individuals have a higher attitude score than others, as shown in Table 3.

Table 7 demonstrates how attitudes toward AI can differ depending on baseline variables. Age groups, Gender, and undergraduate year significantly differ in the proportion of positive attitudes. Undergraduate sixth-year medical students were the most who had a positive attitude 398 (26.4%). More males, 564 (37.4%) had a positive attitude than 463 (30.7%) females. Individuals aged 21–30 had the highest proportion of positive attitudes compared to 1,007 (66.8%) in other age categories.

**Table 7:** Attitude toward AI based on gender, age, qualification level, professional, current status and rank of the doctor.

		**Attitude toward of artificial intelligence**				
		**Negative**		**Positive**		***P*-value**
		**Frequency**	**Percentage**	**Frequency**	**Percentage**	
Age	21–30	445	29.5%	1,007	66.8%	0.013*
	31–40	13	0.9%	12	0.8%	
	41–50	2	0.1%	2	0.1%	
	51–60	4	0.3%	4	0.3%	
	60<	4	0.3%	1	0.1%	
Gender	Male	215	14.3%	564	37.4%	0.001*
	Female	255	16.9%	463	30.7%	
Qualification level	Undergraduate	383	25.3%	869	57.3%	0.086
	Graduate	92	6.2%	163	11.2%	
If undergraduate, then which professional?	1st professional	49	3.3%	77	5.1%	0.000*
	2nd professional	49	3.3%	65	4.3%	
	3rd professional	66	4.4%	79	5.2%	
	4th professional	37	2.5%	45	3%	
	5th professional	98	6.5%	188	12.5%	
	6th professional	78	5.2%	398	26.4%	
	Graduate	98	6.5%	180	11.9%	
If graduate, then current status	Student	401	26.6%	876	58.1%	0.912
	Resident	56	3.7%	126	8.4%	
	Medical practitioner	9	0.6%	15	1.0%	
	Senior house officer	6	0.4%	11	0.7%	
	House officer	3	0.2%	4	0.3%	
If postgraduate, specify the rank:	Student	403	26.7%	881	58.5%	0.659
	Resident	60	4.0%	133	8.8%	
	Senior registrar	5	0.3%	7	0.5%	
	Assistant professor	6	0.4%	6	0.4%	
	Associate professor	0	0.0%	1	0.1%	
	Professor	1	0.1%	4	0.3%	

* *P*-value < 0.05.

The prediction of attitude toward AI among the study sample depending on the demographic variables is given in Table 8. It was observed that the level of undergraduate medical student's 6th year was the only significant factor affecting the attitude toward AI, with *P*-values of less than 0.05. The undergraduate 6th-year students had 2.868 times the attitude toward AI higher than students in the first year. But all other variables were insignificant.

**Table 8:** Binary logistic regression between baseline characteristics of the study population and the attitude toward artificial intelligence^**^.

	**Categories**	***P*-value**	**Odds ratio**	**Lower**	**Upper**
Age	21–30	0.173	Reference		
	31–40	0.073	0.429	0.073	0.429
	41–50	0.344	0.288	0.344	0.288
	51–60	0.440	0.553	0.440	0.553
	60<	0.085	0.138	0.085	0.138
Level of education	undergraduate	Reference			
	graduate	0.458	0.669	0.232	1.931
Gender	Male	Reference			
	Female	0.128	0.835	0.661	1.053
If undergraduate, then which professional?	1st professional	0.000	Reference
	2nd professional	0.379	0.789	0.379	0.789
	3rd professional	0.175	0.710	0.175	0.710
	4th professional	0.224	0.701	0.224	0.701
	5th professional	0.657	1.106	0.657	1.106
	6th professional	0.000	2.868	0.000	2.868
	Graduate	0.548	1.370	0.548	1.370
If Postgraduate, specify the rank	Student	0.584			
	Resident	0.263	1.458	0.263	1.458
	Senior registrar	0.625	1.401	0.625	1.401
	Assistant professor	0.577	0.697	0.577	0.697
	Associate Professor	1.000	-	-	-
	Professor	0.221	5.251	0.370	74.602
Constant	0.002	1.894			

** The logistic regression model was statistically significant, X2(17) = 102.32, p-value = 0.000, Hosmer and lemeshow test: 5.75(P-value = 0.57). The model explained 0.094 Nagelkerke R Square of the variance in the Attitude toward artificial intelligence among doctors and medical students in Syria.

### Practices of AI

The mean score of the practice of AI was 1.90 ± 1.06. Regarding practice of AI, 1,346 (89.3%) had never applied AI in any task of their work, while only 161 (10.6%) had ever practically applied AI. Of 161 who used AI, 95 (60%) declared it easy to apply AI. As expected, most individuals agree that the physician role is essential in the application and evaluation of AI in the medical field, 699 (46.4%). Concerning the practice in the future, 1,184 (78.6%) individuals were ready to apply AI in the future, and 274 (18.2) individuals did not have any opinion on whether or not they would work with AI in the future, As shown in Table 9.

**Table 9:** Descriptive statistics for practice of artificial intelligence.

		**Frequency**	**Percentage%**
Have you ever applied AI technology in any field?	No	1,346	89.3%
	Yes	161	10.7%
Was it easy for you to apply AI?	No	52	3.5%
	never applied	1,360	90.2%
	Yes	95	6.3%
Did AI make your task easy?	No	25	1.7%
	never applied	1,352	89.7%
	Yes	130	8.6%
Do you think physician role is important in application and evaluation of AI in the medical field?	agree	699	46.4%
	strongly agree	531	35.2%
	Don't Know	239	15.9%
	disagree	25	1.7%
	strongly disagree	13	0.9%
Would you like to work on AI in future?	No	49	3.3%
	Don't know	274	18.2%
	Yes	1,184	78.6%

There were significant differences in the practice score as a continuous dependent variable regarding qualification level (*P* < 0.001) and the age groups (P = 0.004), in which the graduate doctors and 41–50-year-old have higher practice score more than others, as shown in Table 3.

Table 10 demonstrates how the practice of AI can differ depending on different baseline variables. Age groups, qualification level, undergraduate year, graduate current status, and postgraduate rank significantly differs in the proportion of good practice except for gender. Individuals aged 21–30 had the highest proportion of good practice compared to other age categories, 207 (13.7%). The undergraduate (9.8%) had a higher proportion of good practice than the postgraduate (5.1%). The 6th-year medical student had the highest proportion of good practice (3%). Graduates considered a student had a higher proportion of good practice than residents and other graduate categories, 152 (10.1). Postgraduate rank regarded as a student had a higher proportion of good practice than a professor and different postgraduate rank categories, 152 (10.1).

**Table 10:** Practice of AI based on gender, age, qualification level, professional, current status and rank of the doctors.

		**Practice of artificial intelligence**				
		**Poor**		**Good**		***P*-value**
		**Frequency**	**Percentage**	**Frequency**	**Percentage**	
Age	21–30	1,245	82.6%	207	13.7%	*0.002
	31–40	16	1.1%	9	0.6%	
	41–50	2	0.1%	2	0.1%	
	51–60	5	0.3%	3	0.2%	
	60<	5	0.3%	0	0.0%	
Gender	Male	676	44.9%	103	6.8%	0.058
	Female	598	39.7%	120	8.0%	
Qualification level	Undergraduate	1,102	72.8%	150	9.8%	*0.000
	Graduate	182	12.5%	73	5.1%	
If undergraduate, then which professional?	1st professional	113	7.5%	13	0.9%	* **0.000**
	2nd professional	98	6.5%	16	1.1%	
	3rd professional	129	8.6%	16	1.1%	
	4th professional	70	4.6%	12	0.8%	
	5th professional	243	16.1%	43	2.9%	
	6th professional	431	28.6%	45	3%	
	Graduate	200	13.3%	78	5.2%	
If graduate, then current status	Student	1,125	74.7%	152	10.1%	*0.000
	Resident	132	8.8%	50	3.3%	
	Medical practitioner	13	0.9%	11	0.7%	
	Senior house officer	10	0.7%	7	0.5%	
	House officer	4	0.3%	3	0.2%	
If postgraduate, specify the rank:	Student	1,132	75.1%	152	10.1%	*0.000
	Resident	138	9.2%	55	3.6%	
	Senior registrar	7	0.5%	5	0.3%	
	Assistant professor	7	0.5%	5	0.3%	
	Associate professor	0	0.0%	1	0.1%	
	Professor	0	0.0%	5	0.3%	

* *P*-value < 0.05.

The prediction of the current practice of AI among the study sample depending on the demographic variables is given in Table 11. It was observed that only the postgraduate rank, specifically the resident and assistant professors, were significant factors affecting the practice of AI with *p*-values < 0.05. The residents and assistant professors had 2.371 and 4.422 times the training of AI higher than students, respectively.

**Table 11:** Binary logistic regression between baseline characteristics of the study population and the Practice of artificial intelligence^***^.

	**Categories**	***P*-value**	**Odds ratio**	**Lower**	**Upper**
Age	21–30	0.845	Reference		
	31–40	0.453	1.476	0.534	4.079
	41–50	0.999	0.000	0.000	.
	51–60	0.300	2.349	0.467	11.817
	60<	0.999	0.000	0.000	.
Level of education	undergraduate	Reference			
	graduate	0.939	1.048	0.311	3.530
Gender	Male	Reference			
	Female	0.076	1.317	0.971	1.786
If undergraduate then which professional?	1st professional	0.400	Reference		
	2nd professional	0.223	1.663	0.733	3.773
	3rd professional	0.538	1.291	0.573	2.906
	4th professional	0.199	1.773	0.739	4.251
	5th professional	0.088	1.844	0.913	3.723
	6th professional	0.664	1.168	0.580	2.352
	Graduate	0.443	1.671	0.451	6.195
If post graduate, specify the rank	Student	0.230		Reference	
	Resident	0.036	2.371	1.058	5.314
	Senior registrar	0.097	3.289	0.805	13.441
	Assistant professor	0.032	4.422	1.141	17.142
	Associate professor	1.000	-	-	-
	Professor	0.999	-	-	-
Constant	0.000	0.082			

*** The logistic regression model was statistically significant, X2 (17) = 81.25, p-value = 0.000, Hosmer and lemeshow test: 18.06 (P-value = 0.012). The model explained 0.094 Nagelkerke R Square of the variance in the Practice of artificial intelligence among doctors and medical students in Syria.

## Discussion

The term “artificial intelligence” (AI) refers to the subfield of computer science that focuses on developing computer programs that can carry out activities that would normally need human intellect (Wahl et al., 2018; He et al., 2019). Due to the fast development of Artificial intelligence in healthcare, it has overcome many problems that arise while delivering medical services (Amisha Malik et al., 2019). Study participants' KAP (Knowledge, Attitude, and Practice) was the focus of this research. In our study, we had 1,494 participants (undergraduate, graduate, and postgraduate medical and dental participants), of which 52% were males. Our findings show that 70% have basic knowledge of AI, 23.7% know AI applications in Medicine, and 34.7% have sufficient knowledge about ML and DL, clearly showing insufficient medical integration knowledge of AI among Syrian participants. Although a thorough knowledge of artificial intelligence is not well observed in the study population, 87.4% of participants recommend its use and find it essential in Medicine. Also, findings report that 68.1% of the Study population showed an important acknowledgment of AI use in the COVID-19 pandemic situation; this percentage is relatively similar and consistent with other studies conducted in Pakistan (74.4%) and the UK (78%), where students acknowledged the essential role of AI in the field of Medicine. Foremother 73.3% of the study population deeply acknowledged the use of AI in radiology which is also committed to the study conducted in Pakistan, where (56.7%) of students and (74%) of doctors considered AI very important in radiology (Sit et al., 2020; Ahmed et al., 2022).

For additional comparing our findings with the results of other studies conducted in many countries and different medical specializations we observed the following: there are connections with a Singaporean study where 67.2% of respondents were keen to get involved in an AI/ML research project; also, in our study, 78.6% of individuals were ready to apply AI in the future. A National Survey Study in Columbia showed that only a minority (29.3%) of respondents agreed AI would replace radiologists in the foreseeable future, and this is consistent with our results, where just (8.4%) strongly agreed, and 97 (13.1%) agreed that AI could replace the physician in the future. In contrast to a Turkish poll, which revealed that just 48.40% of participants had a basic understanding of AI technology, our survey found that (70%) of respondents did. Additionally, there were no links to a German online poll that revealed 52% of respondents were aware of the current debate concerning AI in radiology, but in our research, just 21.2% of participants were aware of this topic. Regarding knowledge of AI and its branches, a cross-sectional study in Ireland showed that 43.4% of participants had not heard of the term ‘machine learning; on the other hand, 65.3% did not know about machine learning in our survey.

In addition, it is worrying that in our study, 21.5% of the study population believe that AI might replace physicians in the future, in contrast to the results found in the Pakistan study, where most participant medical students consider AI as a beneficial healthcare worker aid rather than a potential alternative to physicians, this percentage found in our study is probably caused by misunderstood of AI implications and limitations. Concerning AI incorporation in the medical school curriculum, most participants highly acknowledged the inclusion of the AI curriculum in medical graduate and undergraduate studies, which is also clearly consonant with results found in UK and Pakistan conducted studies (Oliveira et al., 2021; Ahmed et al., 2022).

Overall, results show a relatively moderate basic knowledge of the Syrian participants about artificial intelligence and acceptance of its curriculum inclusion in medical school. Also, Syrian participants showed satisfactory perception and attitude toward AI use in radiology and its importance in diagnosis and healthcare practice. Subtypes, deep AI and its practical implications knowledge seem to be insufficient; this is justified by a traditional curriculum based medical studies with lack or even absence of AI education, its non-integration in medical studies leading to doctors AI disinterest; also, students who did receive AI training are likely more understanding and aware of its limitations that preclude replacement of radiologists, it is also essential to note the significant impact of the Syrian crisis on the educational system and self-learning.

As new science and proper strategic method, AI requires relatively extensive training for physicians, well-established rules and practice ethics, payment restrictions for public or private organizations, and constant upgrades over time, which explains its limited usage globally and current absence in underdeveloped countries such as Syria (Aburas et al., 2018; He et al., 2019; Paranjape et al., 2019). Therefore, we recommend offering training programs, seminars, and webinars about AI, ML, and DL for Syrian health care workers. Also relevant in this study were clinical consequences, knowledge and career ambitions in radiology, and the perspective of AI as a complete replacement for doctors in the future. The findings led us to identify significant deficiencies that need to be addressed, and this necessitates the incorporation, expansion, and enhancement of artificial intelligence (AI) training in medical schools to get rid of erroneous and misleading attitudes and encourage Syrian medical professionals to work in this sector; a basic progression may be achieved by including training on electronic health records (EHRs) into the medical curriculum; in addition, AI training could be provided *via* continuing medical education (CME) programs. Finally, it would seem that the most difficult aspect of implementing AI in healthcare domains is not determining whether or not the technologies will be competent enough to be beneficial; rather, the most difficult aspect is assuring that the technologies will be used in everyday clinical practice (Davenport and Kalakota, 2019; He et al., 2019).

## List of the limitations and the strength

Despite its affordability and usefulness, a cross-sectional research design cannot establish causality at this time. Additionally, by using global sampling and reaching a response rate of 99 %, which is greater than the typical response rate for organizational research surveys, the generalization of this study was improved. Because surveys were anonymous, there was no way to get in touch with respondents after completing the forms to check for unusual responses. Several steps were taken to increase the study's dependability with these limitations. To increase the internal validity of study results, use a validated instrument in addition to controlling for potential confounders in the final model and sample from various research locations. A priori sample size calculations are also performed to ensure the study is robust. Furthermore, the age range of 21 to 30 attracted most participants, leaving the other age groups with little information.

## Conclusions

It is of the utmost importance that immediate steps be taken to enhance the existing condition of the medical professionals and undergraduate medical students in Syria to raise their level of knowledge and influence their attitudes toward using artificial intelligence in medicine. A significant proportion of the respondents said they do not use artificial intelligence in their professional lives. Other than that, many people have a favorable attitude toward its utilization in the medical profession owing to its advantages in enhancing the diagnostic process and how patients are treated. Training medical professionals in Syria to utilize artificial intelligence in their work should be obligatory, and training should occur either online or in person. The Syrian government and international organizations should work together to assist the country's medical professionals.

## Data collection group

1- Rama mazen Reslan: Faculty of medicine, Tishreen University, Latakia, Syria.
2- Nagham Ali Al Hammadi: Faculty of Medicine, Tishreen University, Latakia, Syria.
3- Ahmed Zaher Radwan Radwan: Faculty of medicine, Aleppo University, Aleppo, Syria.
4- Temaa Alkilani: faculty of medicine, Damascus university, Damascus, Syria.
5- Enas Farhan Azzam: Faculty of Medicine, Al-Furat University, Der alzoor, Syria.
6- Fouad Hasib Makhoul: Faculty of Medicine, Al Baath University, Homos, Syria.
7- Ghosoun khazma: Faculty of medicine, University of Kalamoon, Rif dimashq, Syria.

## Data availability statement

The original contributions presented in the study are included in the article/Supplementary material, further inquiries can be directed to the corresponding author.

## Ethics statement

The studies involving human participants were reviewed and approved by all participants could withdraw from the cross-sectional research at any time, and participation was completely voluntary. The participant could not be identified since the study did not provide names or emails. Each participant's identity was therefore wholly protected during the investigation. The University of Aleppo's Ethics Committee granted its permission and gave the research the go-ahead, and the Helsinki Declaration carried out the study. The patients/participants provided their written informed consent to participate in this study. Written informed consent was obtained from the individual(s) for the publication of any potentially identifiable images or data included in this article.

## Author contributions

SSw: conceptualization, data curation, methodology, formal analysis, writing-original draft, and review and editing. HA, NE, MN, MR, AN, BS, MA, EB, LE, SA, EA, ZO, AAls, and SSh: writing—review and final editing. All authors read and approved the final manuscript.

## Conflict of interest

The authors declare that the research was conducted in the absence of any commercial or financial relationships that could be construed as a potential conflict of interest.

## Publisher's note

All claims expressed in this article are solely those of the authors and do not necessarily represent those of their affiliated organizations, or those of the publisher, the editors and the reviewers. Any product that may be evaluated in this article, or claim that may be made by its manufacturer, is not guaranteed or endorsed by the publisher.
