# Supplementary material for: Knowledge, attitude, and practice of artificial intelligence among doctors and medical students in Syria: A cross-sectional online survey
Source: Front Artif Intell. 2022 Sep 29;5:1011524. doi: 10.3389/frai.2022.1011524 (PMC9558737; doi:10.3389/frai.2022.1011524)
Supplement: Supplementary file 1 [file Data_Sheet_1.PDF]

Knowledge, Attitude and Practice of Artificial intelligence among people belonging to Medical field

I declare that I have understood the subject of this research survey and I agree to take part in the survey. I give my permission to have my responses recorded on the survey form. I understand that my responses will be kept strictly confidential and that neither my name nor any other personal information of mine will be identified in any report or communication that uses information from this research. I also understand that if I have any concerns, I may discontinue my participation at any time during the process. I also understand that if I have any concerns, I may contact the research team.

*Required

Demographic Details

1. 1.What is you age group? *

Mark only one oval.

- ⬭ 10 to 20
- ⬭ 21 to 30
- ⬭ 31 to 40
- ⬭ 41 to 50
- ⬭ 51 to 60
- ⬭ above 60

2. 2.Which gender group do you belong? *

Mark only one oval.

- ⬭ Male
- ⬭ Female
- ⬭ Prefer not to say

3. 3. Name of associated Institute? *

4. 4.What is your Qualification level? *

Mark only one oval.

- Undergraduate
- Graduate
- Post Graduate
- Doctorate

5. 5.If Undergraduate then which professional?

Mark only one oval.

- 1st professional
- 2nd professional
- 3rd professional
- 4th professional
- 5th professional

6. 6. If graduate then current status?

Mark only one oval.

- House officer
- Senior House Officer
- Medical Officer
- Medical Practitioner

7. 7. If Post graduate, specify the rank:

Mark only one oval.

- ( ) Resident
- ( ) Senior registrar
- ( ) Assistant professor
- ( ) Associate Professor
- ( ) Professor

8. 8. If applicable, current place of employment

Mark only one oval.

- ( ) BHU
- ( ) RHC
- ( ) DHQ
- ( ) THQ
- ( ) Teaching Hospital

9. 9.Which department do you belong to?

Mark only one oval.

- ⬭ Medical Student
- ⬭ Internal medicine
- ⬭ General Surgery
- ⬭ Radiology
- ⬭ Dermatology
- ⬭ Pathology
- ⬭ Nephrology
- ⬭ Anesthesia
- ⬭ Cardiology
- ⬭ Emergency Medicine
- ⬭ Oncology
- ⬭ Ent
- ⬭ Ophtalmology
- ⬭ Urology
- ⬭ Pedriatrics
- ⬭ Neurology
- ⬭ Gastroenterology
- ⬭ Neurosurgery
- ⬭ Orthopedics
- ⬭ Dentistry
- ⬭ Gyne and obs
- ⬭ Pulmonology
- ⬭ Pharmacology
- ⬭ Other: ____________________

Knowledge of Artificial intelligence

10. 10.Do you know what is artificial intelligence? *

Mark only one oval.

- Yes
- No

11. 11. Do you know about machine learning and deep learning (subtypes of AI)? *

Mark only one oval.

- Yes
- No

12. 12. Do you know about any application of AI in medical field? *

Mark only one oval.

- Yes
- No

13. 13. If yes, then specify:

____

14. 14.Have you ever been taught about Artificial intelligence in medical school? *

Mark only one oval.

- Yes
- No

15. 15.Do you know about application of AI in radiology? *

Mark only one oval.

- Yes
- No

16. 16.Do you know about application of AI in pathology field? *

Mark only one oval.

- Yes
- No

17. 17. If you are a PGR, does your training include curriculum regarding AI?

Mark only one oval.

- Yes
- No

Attitude towards AI

18. 18. Do you believe AI is essential in medical field? *

Mark only one oval.

- strongly agree
- agree
- disagree
- strongly disagree
- no opinion

19. 19. Do you think AI should be included in curriculum in medical school as well as specialist training? *

Mark only one oval.

- Agree
- strongly agree
- disagree
- strongly disagree
- no opinion

20. 20. Do you think that AI aids practitioner in early diagnosis and assessment of severity of disease? *

Mark only one oval.

- strongly agree
- agree
- disagree
- strongly disagree
- no opinion

21. 21. Do you believe that AI will replace physicians in future? *

Mark only one oval.

- Agree
- Strongly agree
- Disagree
- Strongly disagree
- no opinion

22. 22. Do you believe AI is very essential in field of radiology? *

Mark only one oval.

- Agree
- Strongly agree
- disagree
- strongly disagree
- no opinion

23. 23. Do You believe AI is essential in field of Pathology? *

Mark only one oval.

- Agree
- Strongly agree
- disagree
- Strongly disagree
- no opinion

24. 24. Do you think introduction of AI is essential in current Covid 19 pandemic? *

Mark only one oval.

- Agree
- Strongly disagree
- Disagree
- Option 4
- No opinion

25. 25. According to you what might be the reason for reduced practice of AI in Pakistan? *

Tick all that apply.

- [ ] lack of interest
- [ ] lack of awareness
- [ ] lack of proper training
- [ ] lack of curriculum
- [ ] lack of financial resources
- [ ] lack of technological advancement

26. 26. Do you believe AI would be a burden for practitioner? *

Mark only one oval.

- agree
- Strongly agree
- disagree
- strongly disagree
- No opinion

27. 27. Do you believe budget should be allocated for AI to be used in current covid 19 pandemic? *

Mark only one oval.

- agree
- strongly agree
- disagree
- strongly disagree
- no opinion

28. 28. Do you believe AI would increase the percentage of errors in diagnosis? *

Mark only one oval.

- ( ) agree
- ( ) strongly agree
- ( ) disagree
- ( ) strongly disagree
- ( ) no opinion

Practice of AI

29. 29. Have you ever applied AI technology in any field? *

Mark only one oval.

- ( ) yes
- ( ) No

30. 30. which radiographic modalities have you used for AI application? (if applicable)

Tick all that apply.

- [ ] Xrays
- [ ] Ct scan
- [ ] MRI
- [ ] PET scan
- [ ] no any

31. 31. For which pathological assessment u have used AI? (if applicable)

Tick all that apply.

- [ ] Histopathology
- [ ] Culture sensitivity
- [ ] Microscopy
- [ ] Frozen section
- [ ] no any

32. 32. Was it easy for you to apply AI? *

Mark only one oval.

- ( ) yes
- ( ) no
- ( ) never used

33. 33. Did AI made your task easy? *

Mark only one oval.

- ( ) yes
- ( ) no
- ( ) never applied

34. 34. Do you think physician role is important in application and evaluation of AI in medical field? *

Mark only one oval.

- agree
- Strongly agree
- disagree
- Strongly disagree
- no opinion

35. 35.Would you like to work on AI in future? *

Mark only one oval.

- yes
- no
- don't know

This content is neither created nor endorsed by Google.

Google Forms
